# The non-use of evidence in the adoption of a sugar-sweetened beverage tax in OECD countries

**DOI:** 10.1093/eurpub/ckad098

**Published:** 2023-06-16

**Authors:** Johanna Hornung, Fritz Sager

**Affiliations:** KPM Center for Public Management and Multidisciplinary Center for Infectious Diseases (MCID), University of Bern, Bern, Switzerland; KPM Center for Public Management and Multidisciplinary Center for Infectious Diseases (MCID), University of Bern, Bern, Switzerland

## Abstract

**Background:**

Studies confirm the positive effect of sugar-sweetened beverage (SSB) taxation on public health. However, only a few countries in Europe adopt SSB taxes. From a public policy perspective, we investigate the conditions under which countries do or do not follow this evidence.

**Methods:**

Crisp-set Qualitative Comparative Analysis (QCA) of 26 European Organization of Economic Cooperation and Development countries with and without an SSB tax. We test which configurations of conditions (problem pressure, governmental composition, strategic planning, health care system, public health policies, inclusion of expert advice in policymaking) emerge as relevant in determining adoption and non-adoption between the years 1981 and 2021. Pathways that lead to the presence and absence of SSB taxes are identified separately.

**Results:**

At least one of the following configurations of conditions is present in countries that introduced taxation: (i) high financial problem pressure, low regulatory impact assessment activity; (ii) high public health problem pressure, a contribution-financed health care system, no holistic strategy for combatting non-communicable diseases (NCDs); (iii) a tax-financed health care system, a holistic NCD strategy, high strategic and executive planning capacity. In countries that did not adopt SSB taxes, we find (i) high regulatory impact assessment activity, high levels of sugar export; (ii) no holistic NCD strategy, high spending on preventive care; (iii and iv) a lack of strategic planning capacity and either a high share of spending on preventive care or inclusion of expert advice.

**Discussion:**

Evidence inclusion requires clear policy priorities in terms of strategy and resources to promote public health.

## Introduction

To regulate products that have negative effects on public health, governments often employ policy measures. Products like sugar-sweetened beverages (SSB) are detrimental to human health,^1^ and policy measures, like the introduction of a tax,^2^ can help lower the consumption of these products. While there is evidence that a tax on SSBs reduces the consumption and the use of sugar by manufacturers,^3^ only 18 out of 48 countries in the European geographical region have adopted such a tax.^4^ This number includes Norway and Estonia, which once adopted an SSB tax but abolished (Norway) or never implemented it (Estonia). Our research question is: Which conditions lead to the adoption or non-adoption of an SSB tax in European countries?

While evidence for the effectiveness of an SSB tax is publicly available, not all governments follow this evidence-based policy, a common observation in public health policy.^5^ Public policy research provides explanations of why under identical evidence, there is a difference in policy,^6^ which is why we approach this puzzle from a public policy perspective. Kingdon outlines the importance of situational conditions and three streams that must be coupled to achieve agenda shift^7^ and policy change.^8^ The first ‘stream’ of such conditions is related to problem pressure. Problems to which a sugar tax can provide the answer are a disadvantageous economic situation^9^ (financial debt) or severe public health conditions as a consequence of overconsumption of sugar (obesity in particular). Whenever governments experience financial constraints in their public or health care budget, introducing taxation on products that cause them, e.g. by having detrimental effects on public health, holds the potential of generating additional revenue. Problem pressure should therefore be a favourable condition for the adoption of an SSB tax.

The second stream refers to the political factors that enable policy change. It proceeds from the assumption that the policy process is populated by actors with different interests and powers. These can be political parties or lobby groups (e.g. export industry of sugar) advocating against the SSB tax.^10^ The third, policy stream signifies that the instrument to choose must be generally available in the policymakers’ toolkit. For instance, if there already exists either a holistic strategy to combat non-communicable diseases (NCDs) or high shares of expenditures spent on prevention,^11^ this could be relevant to governments’ choosing an SSB tax as an instrument to further foster public health, because they are oriented towards public health policy measures already.

Finally, the streams are shaped by different institutions.^12^ In the case of SSB tax adoption, the inclusion of scientific advice, regulatory impact assessment (RIA) and executive capacity of strategic planning can be considered as relevant institutional conditions. Since SSB taxes are considered an evidence-based policy, expert advisors can deliver scenarios on the positive effect of SSB taxation on public health, thereby fostering its adoption. By contrast, RIAs estimate economic costs of taxation, which could lead to a non-adoption of the tax. Strategic planning capacity is an executive capacity that enables a government’s administration to pursue long-term visions in a sector and execute policy measures effectively. This condition can be supporting or hindering in interaction with other conditions. Finally, it can also make a difference whether the health care system is tax-financed (Beveridge) or contribution-financed (Bismarckian). Since tax-financed health care systems rely on tax revenues to finance health care expenditures, we expect them to be more prone to adopt an SSB tax to cover the negative effects of sugar overconsumption, because their institutional design suggests it.

Understanding the reasons why SSB taxes are adopted or not provides implications for health policymaking because it reveals the conditions under which evidence-based policies are considered in policy instrument selection.^5^ Existing research on SSB tax adoption focuses on either drivers and barriers of the adoption within countries (thereby single cases),^13–15^ or analyses the policy failure of this tax,^16^ or its legal and administrative feasibility^17^ or conducts comparative analyses across countries with a view on specific drivers and barriers, such as narratives and policy learning.^18^ This leaves the need for a systematic comparison of governments’ decisions to adopt an SSB tax.

## Methods

Filling this gap, we heuristically apply Kingdon’s notion of multiple streams^19^ in selecting conditions and, methodologically, make use of the configurative approach of Qualitative Comparative Analysis (QCA).^20^ Hence, our study performs a QCA to identify the conditions under which an SSB tax is adopted and not adopted. We employ QCA because of its ability to compare a limited, medium number of cases (countries) with varying outcomes (SSB tax adopted or not) and conditions. QCA is a configurational method^21^ and builds on Boolean algebra and set theory to identify necessary and sufficient conditions for different outcomes among a definite number of cases. Necessary conditions are those that are present whenever the outcome is present (X ← Y; in set theory: X is a superset of Y). Sufficient conditions are those that whenever present result in the presence of the outcome (X → Y; in set theory: X is a subset of Y).^22^ They are revealed by a process of logical minimization through a comparison of cases with their configurations of outcomes and conditions. QCA proceeds from the premises of equifinality (many pathways to the same outcome), multifinality (the same condition can lead to different outcomes depending on the combination of conditions) and asymmetry (conditions leading to the presence of an outcome do not automatically lead to its absence when absent). The method has proven its ability to generate insightful results in comparative analyses of policy adoption.^23^^,^^24^

Our analysis considers the problem stream (high levels of obesity and a country’s debt), policy stream (NCD strategies and prevention), political stream [power of interest groups (via the levels of exported sugar)] and institutions [the health care system, the extent of executive strategic planning, the inclusion of expert advice (in RIAs and as strategic capacity)] as potential conditions for the adoption and non-adoption of an SSB tax. It follows the standards of good practice for conducting a QCA^22^ by beginning with the identification of necessary conditions and proceeding with testing the sufficiency of conditions.

### Case selection

We select the European members of the Organization of Economic Cooperation and Development (OECD), a total case number of 26. The case selection is justified by the research interest to tease out the relevance of conditions for the introduction of SSB taxes while keeping constant the frame conditions of industrialization, economic situation, culture and democratic quality. We included OECD countries to include all countries in the European region so that they are not influenced by EU policy on national policymaking. Data on (the type of) an existing SSB tax are provided by the Obesity Evidence Hub^25^ and the Global SSB tax database.^4^ Twelve (*N* = 12) European OECD countries have an SSB tax: Belgium, Estonia, Finland, France, Hungary, Ireland, Latvia, Norway, Poland, Portugal, Spain and the UK. Estonia, Norway and Spain are special cases, though: Estonia and Norway adopted SSB taxes but never implemented or abolished them, respectively. In Spain, an SSB tax first only existed at the subnational level, but a value-added tax has been introduced nationwide. The decision to adopt a tax is the relevant information for the analysis. Fourteen (*N* = 14) never adopted an SSB tax: Austria, Czech Republic, Denmark, Germany, Greece, Iceland, Italy, Lithuania, Luxembourg, the Netherlands, Slovakia, Slovenia, Sweden and Switzerland.

### Calibration of outcome

Whether an SSB tax exists (1) or not (0) is a straightforward calibration. While there are distinctions between different types of SSB taxes,^26^ we are interested in the conditions under which any of them is adopted or not, which allows for a clear dichotomous calibration. Since the outcome is dichotomous, crisp-set QCA must be chosen instead of fuzzy-set QCA.

### Calibration of conditions

A calibration of the conditions must consider that each country’s adoption of an SSB tax took place at different points in time. For the countries with an SSB tax, we perform the operationalization of conditions for the point in time 2 years prior to the policy adoption. For all other countries, we calculate the conditions as the mean over the years of our study period (1981–2021). Doing so offers several advantages. First, the operationalization allows for a comparative assessment of the (presence or absence of the) outcome and the conditions that potentially led to it. Second, the conditions are quite stable over the years so the calibration of the dichotomous condition does not vary substantially.

An SSB tax can address the challenges of a poor economic situation or severely negative public health conditions brought about by overconsumption of sugar. We operationalize these conditions by measuring substantial levels of financial debt (DEBT) and high numbers of people suffering from obesity (OBES).

For an SSB tax to be adopted, it is generally necessary for policymakers to have this policy instrument available in their toolkit. Our analysis captures this condition by measuring the ‘existence of an operational, multisectoral national NCD policy, strategy or action plan that integrates several NCDs and their risk factors’^27^ (HOLNCD). We also calibrate the share of health care expenditures devoted to prevention (PREV) to depict the general orientation towards public health in financial terms.^28^

We measure the inclusion of scientific evidence via the application of regulatory impact assessments (RIAP) and the extent to which expert advice is considered in government (ADV) using the annual expert survey of the Sustainable Governance Indicators (SGI).^29–31^ SGI indicators’ values range from 1 to 10, and 6 is the qualitative threshold used in QCA studies.^32^ These values are also taken as thresholds for the existence or absence of the condition. We calibrate health care system institutions (BEV) by measuring whether health insurance is mainly tax-financed (=1) or contribution-financed (=0).^33^ We operationalize the institutions of strategic planning (STRAT) using the above-mentioned SGI data. The power of interest groups is measured via a proxy: the extent to which sugar is exported by a specific country (EXP).

Table 1 lists the outcome (SSB tax or not) and all conditions examined with their operationalization and data source.

**Table 1:** Calibration and data sources of outcome and conditions

Condition	Description and measurement	Data source	Calibration
TAX	Existence of SSB tax (OUTCOME)	Obesity Evidence Hub^25^ and Global SSB Tax Database^4^	Yes (1), no (1)
DEBT	Substantially high financial debt	World Bank^38^	Threshold: 80
OBES	High share of people suffering from obesity	World Health Organization (WHO)^39^	threshold: mean of 18% over the years
EXP	High export rates of raw sugar (or equivalent), divided by population	Food and Agriculture Organization of the United Nations (FAO)^40^	Threshold: 25,000 tonnes per capita
STRAT	Strategic capacity: strategic planning	Sustainable Governance Indicators (SGI)^30^	Threshold: 6
BEV	Health Care System (Beveridge)	Schubert, de Villota and Kuhlmann^33^	Yes (1), no (1)
HOLNCD	‘Existence of an operational, multisectoral national NCD policy, strategy or action plan that integrates several NCDs and their risk factors’	WHO^27^	Yes (1), no (1)
PREV	Share of health expenditures spent on prevention	Organization for Economic Cooperation and Development (OECD)^28^	Threshold: 0.3 %
RIAP	Evidence-based instruments: RIA application	SGI^29^	Threshold: 6
ADV	Strategic capacity: expert advice	SGI^31^	Threshold: 6

## Results

### Necessity analysis: conditions for SSB tax adoption

The analysis reveals that no single condition emerges as necessary for adopting an SSB tax. However, the existence of debt is part of several necessary configurations combined with the logical ‘or’ (either one or the other condition, including but not limited to the combination of the two, is necessary for the outcome to occur). For example, whenever an SSB tax is present, there is a high amount of debt or a weak sugar industry (indicated by a low export volume of raw sugar or equivalent; with a consistency value of 1, a coverage value of 0.632 and a relevance of necessity value of 0.500). However, configurations with logical ‘or’ are difficult to fully interpret when performing a necessary condition analysis because they tend to become trivial without a sound theoretical argument behind them. In this case, we can at least conclude that economic considerations seem to be relevant to SSB tax adoption.

### Sufficiency analysis: conditions for SSB tax adoption

**Figure 1:**
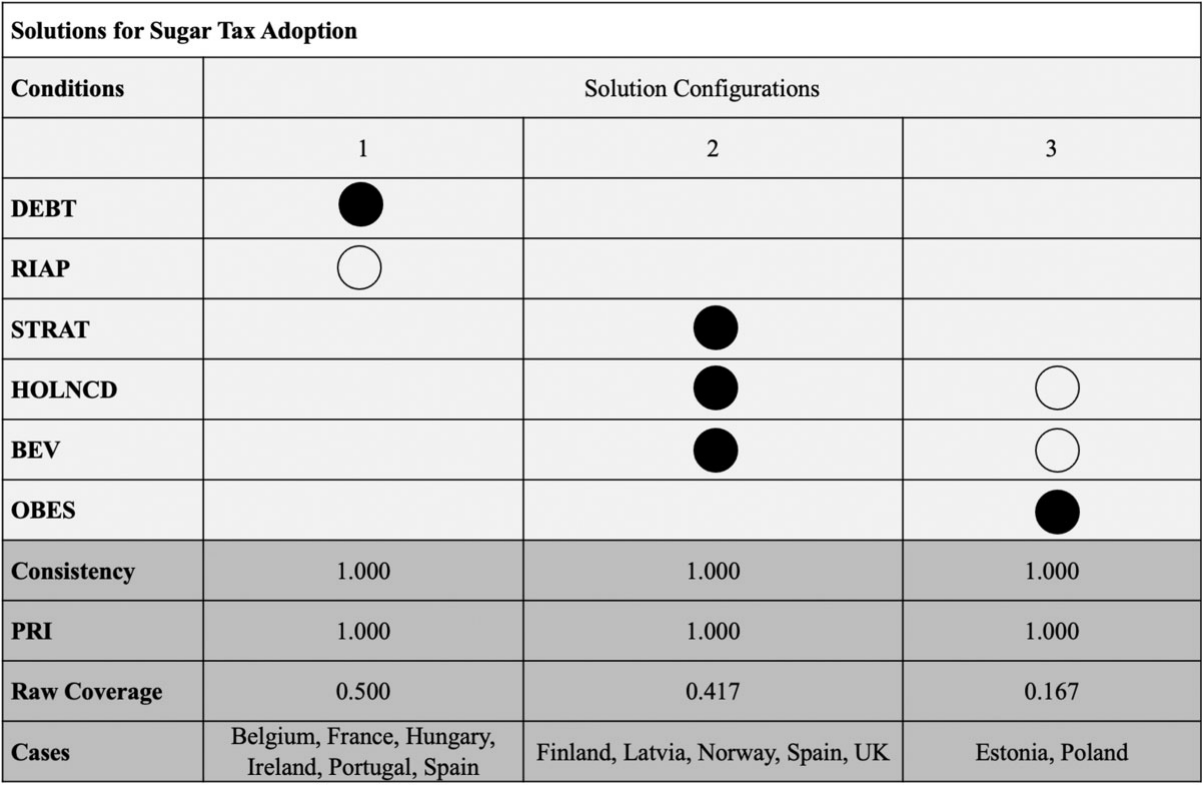
Sufficient condition for sugar tax adoption. Source: Authors’ table. Full circles depict the presence of the condition. Empty circles depict the absence of the condition. The consistency value indicates to what extent the condition always leads to the same outcome (in this case the adoption of a sugar tax), or whether it produces inconsistencies (i.e. sometimes the presence and sometimes the absence of a sugar tax). The parameter indicating the proportional reduction in inconsistency (PRI) expresses whether the (configuration of) condition(s) is a subset of the presence of the outcome (but not of the absence of the outcome) and—the higher—indicates a high relevance of the identified configuration in being sufficient for the outcome of a sugar tax. The coverage value indicates how many cases are covered by the respective configuration of conditions

The analysis of sufficiency identifies three pathways to the outcome of an SSB tax. Figure 1 depicts the results derived from a truth table analysis with a minimization procedure showing the parsimonious solution. All pathways have a consistency value of 1, which means that their presence is always connected with the observation of the outcome of SSB tax adoption, and there is no case in which the pathway is present, but the combination is absent.

In half of the cases with SSB tax, there is a combination of substantial financial debt and no RIA, which is identified as sufficient for the outcome to occur. Belgium, France, Hungary, Ireland, Portugal and Spain show this combination of conditions.

The second and third pathways for explaining the outcome of SSB tax adoption both include the conditions of a holistic NCD policy strategy and a tax-financed health care system. However, these conditions can lead to the adoption of an SSB tax when present or when absent depending on their configuration with another condition. If both conditions are present, the combination with a high level of strategic planning in the executive branch of government favours the SSB tax as a policy instrument. This pathway explains the existence of an SSB tax in Finland, Latvia, Norway, Spain and the UK. On the other hand, if there is a contribution-based health care system and no holistic strategy, the problem of obesity triggers the adoption of an SSB tax (observed in Estonia and Poland).

### Necessity analysis: conditions for the non-adoption of an SSB tax

To examine the necessary and sufficient conditions for the non-adoption of SSB tax, this section reiterates the process of analysis for the negative outcome (no SSB tax). When analyzing necessary conditions, it becomes clear that the non-adoption of SSB tax is observed whenever there is no financial problem (consistency value: 0.929; coverage value: 0.722; relevance of necessity: 0.615). This supports the argument that the absence of an economic problem is necessary for the non-adoption of an SSB tax, although it alone is not sufficient for an SSB tax adoption.

### Sufficiency analysis: conditions for the non-adoption of an SSB tax

**Figure 2:**
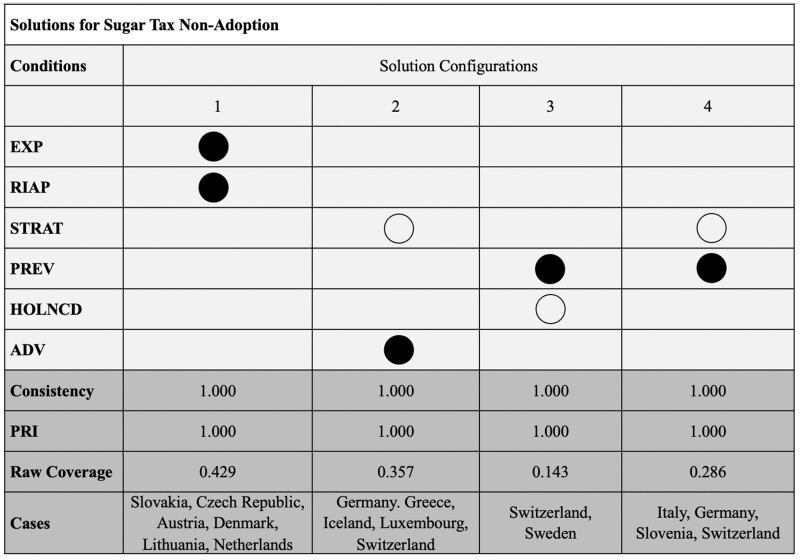
Sufficient condition for sugar tax non-adoption. Source: Authors’ table. Full circles depict the presence of a condition. Empty circles depict the absence of a condition. The consistency value indicates to what extent the condition always leads to the same outcome (in this case the non-adoption of a sugar tax), or whether it produces inconsistencies (i.e. sometimes the presence and sometimes the absence of a sugar tax). The parameter indicating the proportional reduction in inconsistency (PRI) expresses whether the (configuration of) condition(s) is a subset of the absence of the outcome (but not of the presence of the outcome) and—the higher—indicates a high relevance of the identified configuration in being sufficient for the non-adoption of a sugar tax. The coverage value indicates how many cases are covered by the respective configuration of conditions

Figure 2 shows the sufficient configurations of conditions for the non-adoption of SSB taxes. When looking at the pathways that lead to non-adoption, the first pathway depicts that the presence of an RIA in conjunction with the presence of high shares of the export of raw sugar (or equivalents) explains the non-adoption of the SSB tax. This pathway alone explains 43% of the cases without an SSB tax, namely Slovakia, Czech Republic, Austria, Denmark, Lithuania and the Netherlands.

Strategic planning is a condition that occurs in two of the three configurations. It contributes to the adoption of the SSB tax when present and contributes to its non-adoption when absent. The combination of an absence of governmental strategic planning with expert advice or high spending on prevention results in the non-adoption of an SSB tax. This seems to be true of the countries of Germany, Italy, Slovenia and Switzerland. The inclusion of expert advice combined with the lack of strategic planning therefore leads to the non-adoption of SSB tax. Like the previous pathway, the inclusion of expert advice does not help in evidence-based policymaking if it is not combined with strategic planning and the capacity to pursue a governmental strategy. Greece, Iceland, Luxembourg, Germany and Switzerland fall under this explanatory pattern.

In the final pathway, a high share of spending on prevention is part of a sufficient condition for the non-adoption of an SSB tax. This time spending on prevention is combined with the lack of a holistic NCD strategy. Sweden and Switzerland depict this configurational condition.

## Discussion

Based on our results, we found that countries apparently do not adopt an SSB tax because of the evidence that it is effective, but because of other conditions. RIA plays a role only if it is absent. While this may seem surprising at first, the result makes sense given the neoliberal rationale of RIA: It commonly focuses on the economic consequences of new regulations for business.^34^ This perspective generally leads to negative assessments of new taxes and may result in an overestimation of the negative impact of SSB taxes on consumption with negative financial consequences for retailers. This potentially explains why the adoption of SSB tax was observed in countries that have a low RIA activity and a weak financial situation that they address with additionally expected tax revenues.

A holistic strategy of fighting NCDs and a tax-financed health care system are sometimes present and sometimes absent parts of a configuration that explains the outcome of SSB tax adoption. Although this connection seems paradoxical at first glance, it can be theoretically justified: It makes sense that tax-financed health care systems with a public health strategy that is already in place adopt an SSB tax if they have the strategic planning capacity to do so. Recent research on design paths shows that policy design choices often follow the inherent institutional designs^35^ and tax-financed health care systems steer through taxation. In Bismarckian health care systems, SSB tax adoption rather seems to be a response to high obesity levels, arguably because such high levels combined with the absence of a public health strategy strain the financial resources of sickness funds. Bismarckian systems are designed to keep insured individuals healthy because negative health consequences will be financed by sickness fund contributions. If obesity levels increase, sickness funds might have an interest in alternative modes of financing such expenses and support the introduction of an SSB tax.

While the inclusion of evidence in policymaking through expert advice or RIA does not contribute to the adoption of SSB tax, it is also not possible to trace the non-adoption of an SSB tax to the non-consideration of evidence (the inverse outcome to the inverse condition). It seems that whenever evidence is included, SSB taxes are not adopted: In countries with high RIA and high export shares for sugar, SSB tax adoption was observed. Substantial export values indicate a large industry behind the production of raw sugar that contributes to economic affluence. An RIA in place suggests a negative view on introducing an SSB tax in light of a neoliberal consideration of evidence connected to this policy instrument. As a result, the conditions together form a configuration that yields a lack of support for an SSB tax out of liberal economic reasons. An RIA, originally introduced to foster evidence-based policymaking, seems to have the opposite effect.

Furthermore, in countries with expert advice included or high spending on prevention, and with the absence of strategic planning, SSB non-adoption is observed. This can theoretically be explained: If there is expert advice provided but not incorporated in a strategic planning unit, it will not be systematically considered in policymaking. At the same time, if governments are already investing heavily in preventive care in a rather unsystematic way and without strategic planning, there may be insufficient consideration given to the evidence of the effect of an SSB tax. A high share of spending on prevention and a lack of a holistic strategy NCD strategy seems to have the same outcome: High but—because of a lacking strategy—unsystematic spending on health prevention does not favour an SSB tax.

The findings challenge the often-assumed mechanism between the integration of expert advice as a process feature that fosters the outcome of evidence-based policymaking. Integrating policy advice in policymaking (through RIA or expert advice) may even hinder the consideration of evidence in policy adoption. To make governments consider evidence in policy adoption, the integration of policy advice needs to be combined with other conditions. In sum, the findings indicate that politics trumps evidence in policymaking.

Despite the theoretically sound and empirically enlightening results, the study underlies several limitations. Firstly, because QCA does not allow for including a temporal component in its analysis, it is by default cross-sectional. While this corresponds to our research interest in identifying conditions that exist when an SSB tax is adopted or not adopted, it is important to further investigate the reasons for updates in taxation design and abolishment or non-implementation. This is especially related to a second limitation that concerns the special country cases of Estonia, Norway and Spain. All three countries were included in the group of countries that have adopted an SSB tax, because they did. However, Estonia adopted one that was never implemented, and Norway abolished its SSB tax in 2021.^36^ This poses an interesting case for examining the conditions under which adopted public health policies are not implemented or abolished. Spain only had a subnational tax in Catalonia for many years, before introducing a VAT tax on sugar-sweetened products. Thereby, it poses an exemplary case for investigating the federal dynamics of public health policymaking and its effect on tax design. We encourage further research in these regards.

The results provide important conclusions. First, they confirm existing research that outlines the context-dependence of factors driving commitment towards improving nutrition.^37^ Second, there is no sign that institutional and structural attempts to integrate advice into policymaking foster evidence-informed policymaking, which leaves us with the challenge of how to ensure that evidence is considered in the policy process, especially in such vital areas as public health policymaking. A look at the conditions that contribute to the adoption of an SSB tax suggests that to successfully pursue an evidence-informed policy, practitioners should combine evidence with both a problem and a holistic strategy to embed it. The analysis suggests that these need to be embedded in structures that ensure the strategic capacities of governments and administrations to realize the suggested policies. If expert advice, or arguably evidence-based procedures such as RIA, is only included in the policy process without strategy and executive resources, it may even be detrimental to evidence-based policy.

## Data Availability

The data underlying this article are available in the figshare repository at https://www.doi.org/10.6084/m9.figshare.20331690. Including expert advice in policymaking does not necessarily lead to the adoption of public health policies that evidently improve public health outcomes. Strengthening the executive capacity of strategic planning in governments can foster the adoption of sugar-sweetened beverage taxes and public health policies in general. Regulatory impact assessment and a strong sugar export industry hinder the adoption of sugar-sweetened beverage taxes. There is no straightforward connection between the adoption of a sugar-sweetened beverage tax, and a holistic NCD strategy and spending on prevention.
